# Patient reported physical function, mental health, and treatment patterns in dermatomyositis: survey results from a cross-sectional study of adult dermatomyositis patients

**DOI:** 10.1186/s41927-025-00458-2

**Published:** 2025-02-24

**Authors:** Lisa Christopher-Stine, Julie J. Paik, Alexandra S. Goriounova, Paul N. Mudd

**Affiliations:** 1 Division of Rheumatology, Department of Medicine, Johns Hopkins University School of Medicine, 5200 Eastern Avenue; Mason F. Lord Center Tower #4500, Baltimore, MD 21224 USA; 2 Priovant Therapeutics Inc, New York, NY USA

**Keywords:** Dermatomyositis, Quality of life, Survey, Burden of disease, Corticosteroids, Opioids

## Abstract

**Objectives:**

Dermatomyositis (DM) is a rare and progressive immune-mediated disease with no cure and significant patient burden that encompasses physical, mental, and financial impacts. Patients experience debilitating symptoms that may include muscle weakness, itchy and painful rash, joint pain, and fatigue. Despite the heterogeneity of the disease and the breadth of possible symptoms, the impact of DM on a diverse range of patients’ quality of life (QoL) has not been well-characterized in literature. The aim of this study was to describe the experiences of patients living with DM as they relate to physical and mental impacts, productivity, and treatment patterns and satisfaction.

**Methods:**

To address this deficiency, a 60-question survey was developed to capture adult patient perspectives on the impact of DM on their QoL. Members of The Myositis Association (TMA) with a self-reported diagnosis of DM who were 18–75 years old and whose disease duration was ≥ 1 year were invited to complete the online survey.

**Results:**

Respondents were predominantly female (88%, 172/195), white (82%, 160/195), and had a median age of 57 years. Approximately 50% (98/195) of the respondents rated their overall symptoms as moderate and the three most bothersome symptoms were muscle weakness (44%, 86/195), fatigue (43%, 84/195), and muscle pain (30%, 59/195). Almost all respondents (83%, 162/195) experienced some form of mental stress due to DM and reported that this had a negative impact on interpersonal relationships. The majority (87%, 170/195) of respondents were less than satisfied with the level of support they received for DM.

**Conclusions:**

Our study demonstrates the significant burden of DM on a patients’ QoL and there remains a large unmet need for financial support, mental health care, and improved treatment options for patients living with DM.

**Supplementary Information:**

The online version contains supplementary material available at 10.1186/s41927-025-00458-2.

## Introduction

Dermatomyositis (DM) is a rare, progressive, and debilitating type of idiopathic inflammatory myopathy that has a profound impact on patients’ quality of life (QoL) [1, 2]. Symptoms may include proximal muscle weakness and pain, itchy and painful skin manifestations (e.g., Gottron’s papule and heliotrope rash), fatigue, and joint pain and stiffness. Patients experience one or more of these symptoms in varying degrees of severity. Patients with DM are also more likely to have a variety of comorbidities, including interstitial lung disease, gastrointestinal complications, cardiovascular disease, and malignancy [3–6]. As a result, DM patients are likely to experience negative impacts on various aspects of daily life, including completing daily tasks, social and mental health, and financial wellbeing. Recently, studies have demonstrated that DM patients have increased medication burden, inpatient admissions, and loss of working hours due to disability [7–10]. Additionally, there remains a lack of a targeted therapy for DM patients, presenting a significant unmet need and difficulty with symptom management [11, 12]. Due to the clinically heterogenous manifestation of DM in both presentation and severity, understanding the diverse DM patient perspective is important for researchers, clinicians, policy makers, patient advocates, and patients themselves. In a recent survey-based cross-sectional study of untreated depression and anxiety in cutaneous lupus and DM patients, 43.9% of DM patients met the criteria for anxiety or depression requiring treatment [13]. In another survey-based study of the impact of grip strength on QoL in DM and polymyositis patients, low grip force had a strong negative impact on vitality and mental health as measured by the Short Form-36 [14]. However, a more comprehensive assessment of the impact of DM and treatment patterns on physical, social, mental and financial QoL are not yet well-characterized in literature. To address this deficiency, a comprehensive survey was developed that aimed to capture a broad patient experience covering both the physical and psychosocial burdens of the disease.

## Methods

### Questionnaire

The web-based survey was developed on the SurveyMonkey platform (www.surveymonkey.com) and contained 6 screening questions and 60 survey questions (see full questionnaire in Additional File 1 Data S1). The screening questions were made up of yes/no questions (*n* = 4) and multiple-choice questions (*n* = 2). The survey included a variety of questions, including Likert scale questions (*n* = 24), multiple-choice questions (*n* = 22), yes/no questions (*n* = 6), number-entering questions (*n* = 4), dropdown questions (*n* = 3), and an open-ended question (*n* = 1). The survey was expected to take no more than 15 min to complete.

The dermatomyositis-focused questions were for the most part developed or derived from modifying items from several patient-reported outcome measures to cover the following areas of DM impact:

- The most bothersome symptoms and their severity, assessed through a targeted review of the literature and qualitative interviews with DM patients.
- Impact on sexual desire, adapted from the Female Sexual Function Index [15].
- Intimacy impact, adapted from the Systemic Lupus Erythematosus Fatigue, Activity participation, Mental health, Isolation, Love and intimacy, and You/fulfilling family roles questionnaire [16].
- Mental health impact, adapted from the Patient-Reported Outcomes Measurement Information System (PROMIS) Anxiety Short Form and the PROMIS Depression Short Form [17].
- Career impact, adapted from the Work Productivity and Activity Impairment – General Health [18].
- Institutional/organizational support.
- Treatment preference.
- General impact of dermatomyositis.

The most bothersome symptoms of DM were identified via prior qualitative interviews with two focus groups of patients with DM (*N* = 14). During the qualitative interviews, patients were asked to talk about their most bothersome symptoms and the concepts and issues that were meaningful and important to them when describing their DM experience. Content analysis was then conducted to synthesize the qualitative data gathered from the interviews. Next, important items and concepts elicited by the patients during the qualitative interviews were selected and put into question form. These questions were then cognitively debriefed with five respondents who had been diagnosed with dermatomyositis for three to eight years, had an average age of 54.6 years and were 60% female. Standard “think aloud” and “verbal probing” procedures were used. Patients were asked first to respond to all the items, one at a time. This was followed by a review of each of the questions one-by-one by the interviewer in order to determine (1) if the questions were easily understood by all education levels, (2) if the questions were easy to complete, (3) if the questions and their responses were relevant to patient’s experience of disease, and (4) if there were preferences regarding item language, phrasing, and type of response options. The questions were revised based on the cognitive debriefing interviews. The questions were also reviewed by a DM physician for relevance and significance.

### Data collection

The survey protocol received an Institutional Review Broad (IRB) exemption on June 22, 2022, from the Western IRB-Copernicus Group (WCG) prior to survey conduct. Participants were recruited via The Myositis Association (TMA email member list). Electronic consent to collect, analyze, and publish results from the survey was obtained from every participant prior to them completing the survey. The participant’s information was kept completely confidential to the full extent of the law. Responses were recorded anonymously and assigned numerical identifiers. Contact information (email address and mobile phone number) was only collected at the end of the survey in order to electronically distribute honorarium and was not provided to the sponsor. The participant’s contact information was not linked to their survey responses and was deleted upon successful receipt of the honorarium.

To be eligible to participate in this online survey, TMA members had to meet the following inclusion criteria:

1. 18–75 years of age, inclusive
2. Self-reported diagnosis of DM made by a health care provider (e.g., doctor, nurse practitioner, physician assistant)
3. Currently experiencing muscle, skin, or other symptoms due to DM.
4. Have been experiencing DM symptoms for at least one year
5. Able to provide informed consent.

The survey was designed so that unanswered questions were not permitted, except for three optional questions. Completed surveys were anonymous and assigned numerical identifiers. An email invitation to participate in the online survey along with the survey link was sent by TMA to its U.S. adult members who self-identified as having a diagnosis of DM. The online survey was launched on August 12, 2022, and remained open to participation through October 3, 2022.

Data are presented here using descriptive statistics. Continuous variables were presented using means and categorical variables were presented using frequencies and percentages. Chi-squared tests were used to analyze differences across the groups that were stratified post hoc by disease severity (mild vs. moderate/severe/very severe) or by current OCS use (yes vs. no); A two-sided P-value of < 0.05 was considered statistically significant and each test between the two groups was a unique hypothesis, but statistical significance using the Bonferroni correction for multiple comparisons was also conducted as a sensitivity analysis.

The reliability and internal consistency of the survey was assessed using Cronbach’s Alpha. The survey demonstrated excellent overall reliability (Cronbach’s alpha = 0.94). See Supplementary Table S1 for Cronbach’s alpha of individual domains.

## Results

### Patient characteristics

**Fig. 1:**
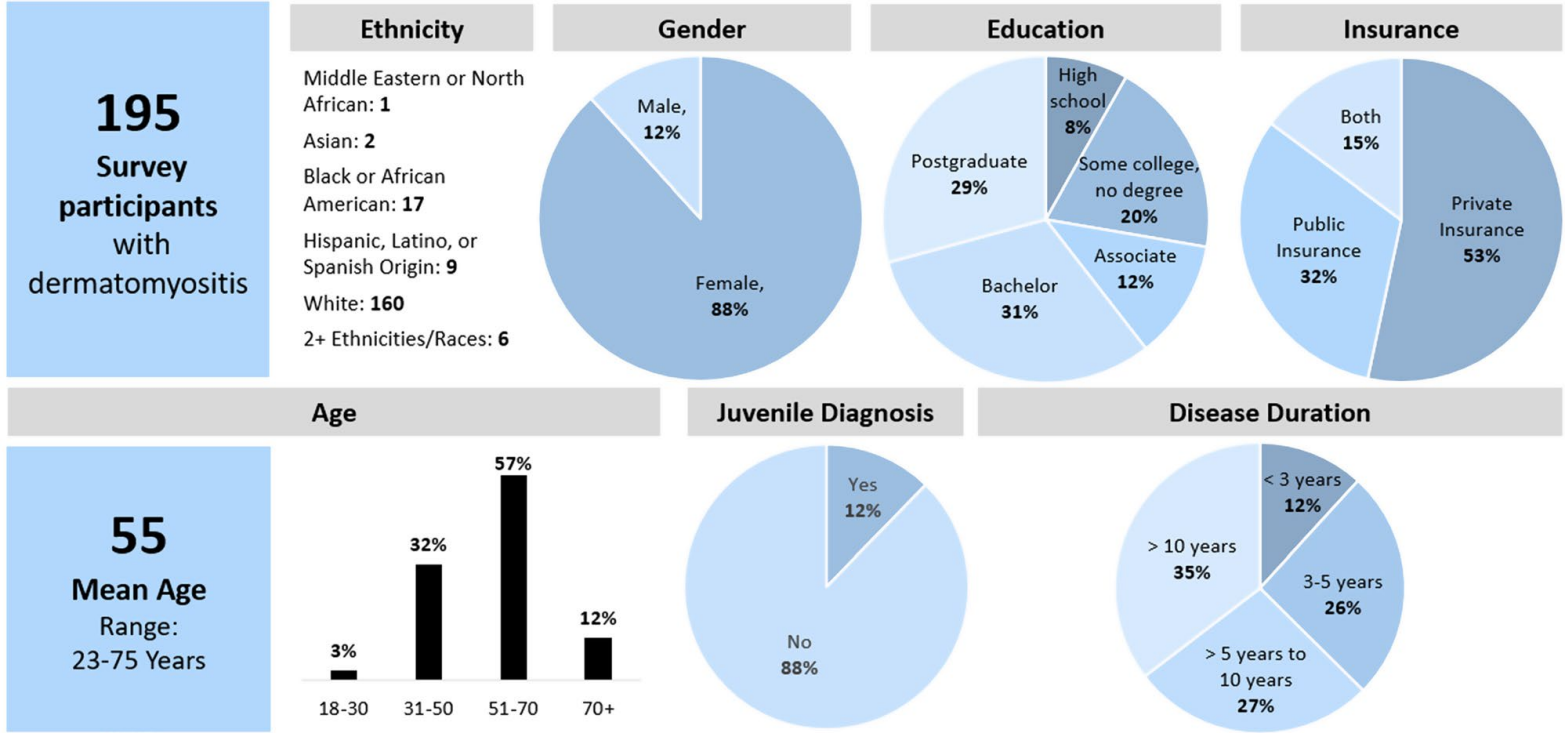
Survey respondent demographics

The survey was fully completed by 195 eligible respondents. The characteristics of the 195 respondents are shown in Fig. 1. The median age was 57 years [interquartile range (IQR): 45, 65] and 88% self-reported as female. Respondents resided in 40 States. The state with the highest representation was California (10%, 20/195), followed by New Jersey, New York, Pennsylvania, and Florida (all 7%, 14/195). When asked to choose one ethnicity/race with which they most closely identified, 82% (160/195) self-identified as White, 9% (18/195) as Black or African American, 5% (10/195) as Hispanic, Latino, or Spanish Origin, and 3% (6/195) as mixed race. In terms of education, 31% (60/195) had a bachelor’s degree, 29% (57/195) had a postgraduate degree, 20% (39/195) had some college, 12% (23/195) had an associate degree and 8% (16/195) had a high school education.

All respondents had some form of health insurance coverage. Most of the respondents (68%, 133/195) reported having private/commercial health insurance, followed by 47% (92/195) with public/government health insurance (e.g., Medicare, Medicaid), and 15% (29/195) with both private and public health insurance. In terms of DM symptom duration, 35% (68/195) reported experiencing symptoms for more than 10 years, 27% (53/195) reported > 5 years to < 10 years, 26% (51/195) reported 3 to 5 years, and 12% (23/195) reported less than 3 years. Most respondents were diagnosed by a rheumatologist (65%, 127/195) or a dermatologist (27%, 53/195). Only 12% (23/195) reported being diagnosed with juvenile DM in the past.

### Burden of disease

**Fig. 2:**
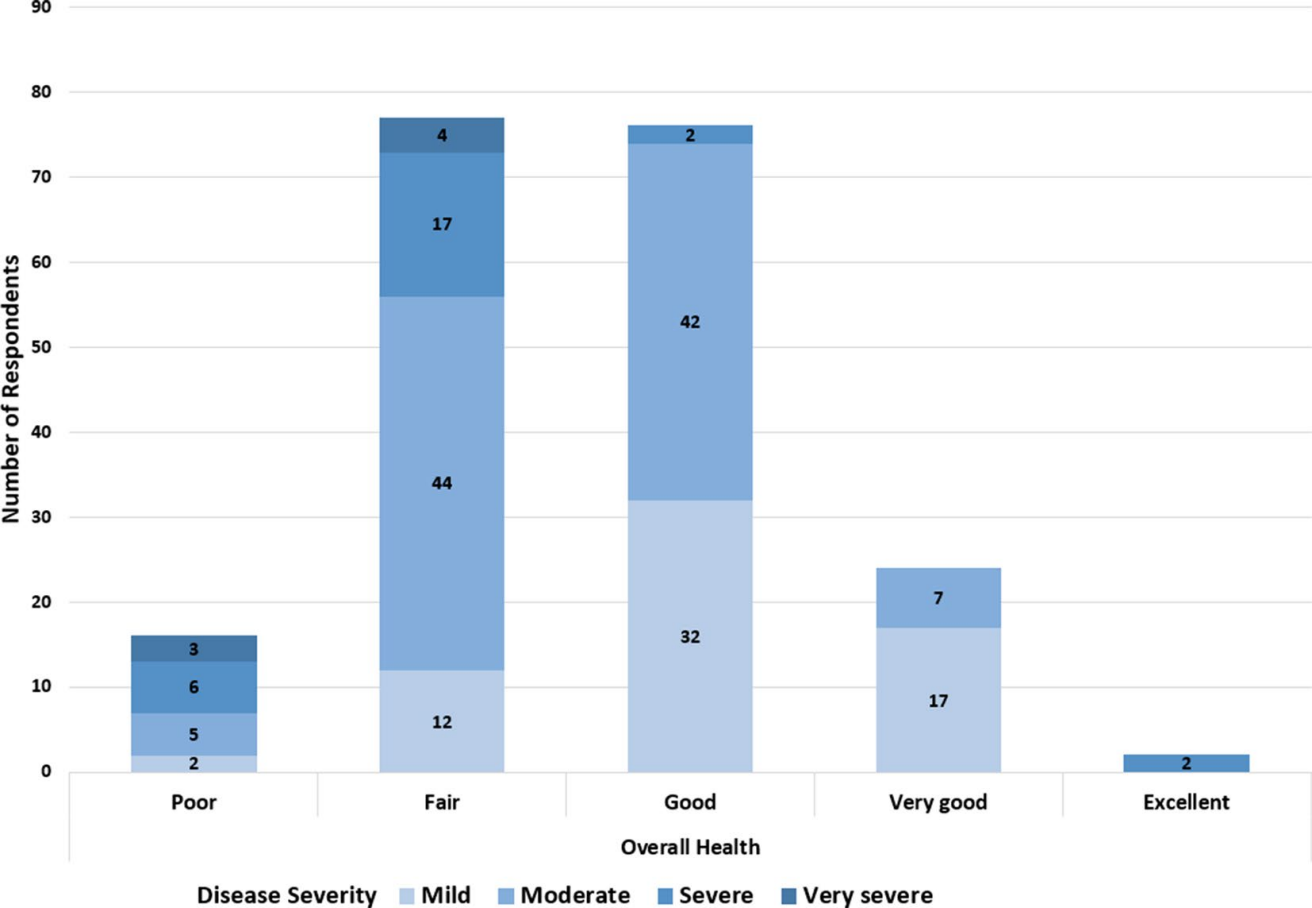
Overall Health vs. DM Symptom Severity. Most respondents categorized their overall health as “fair” or “good”, and their symptom severity as “moderate”. The legend refers to the classification of DM symptom severity. There was a total of 195 respondents

When asked to describe the current severity of their overall DM symptoms, approximately 50% (98/195) of the respondents rated their overall symptoms as moderate, followed by 33% (63/195) who rated their symptoms as mild, 14% (27/195) as severe, and 4% (7/195) as very severe. In general, increased severity of DM symptoms had a negative impact on overall health (Fig. 2). Respondents who described the severity of their symptoms as “severe” or “very severe” also reported lesser health and did not rate their overall health any higher than “good”. In fact, respondents with very severe symptoms rated their overall health as only “poor” or “fair”. Respondents with moderate, severe, or very severe symptoms were significantly more likely to rate their health as poor or fair than those with mild symptoms (*p* < 0.0001). Conversely, respondents who reported severity of their DM symptoms to be mild were significantly more likely to rate their health as good, very good, or excellent (*p* < 0.0001).

#### Bothersome symptoms

**Fig. 3:**
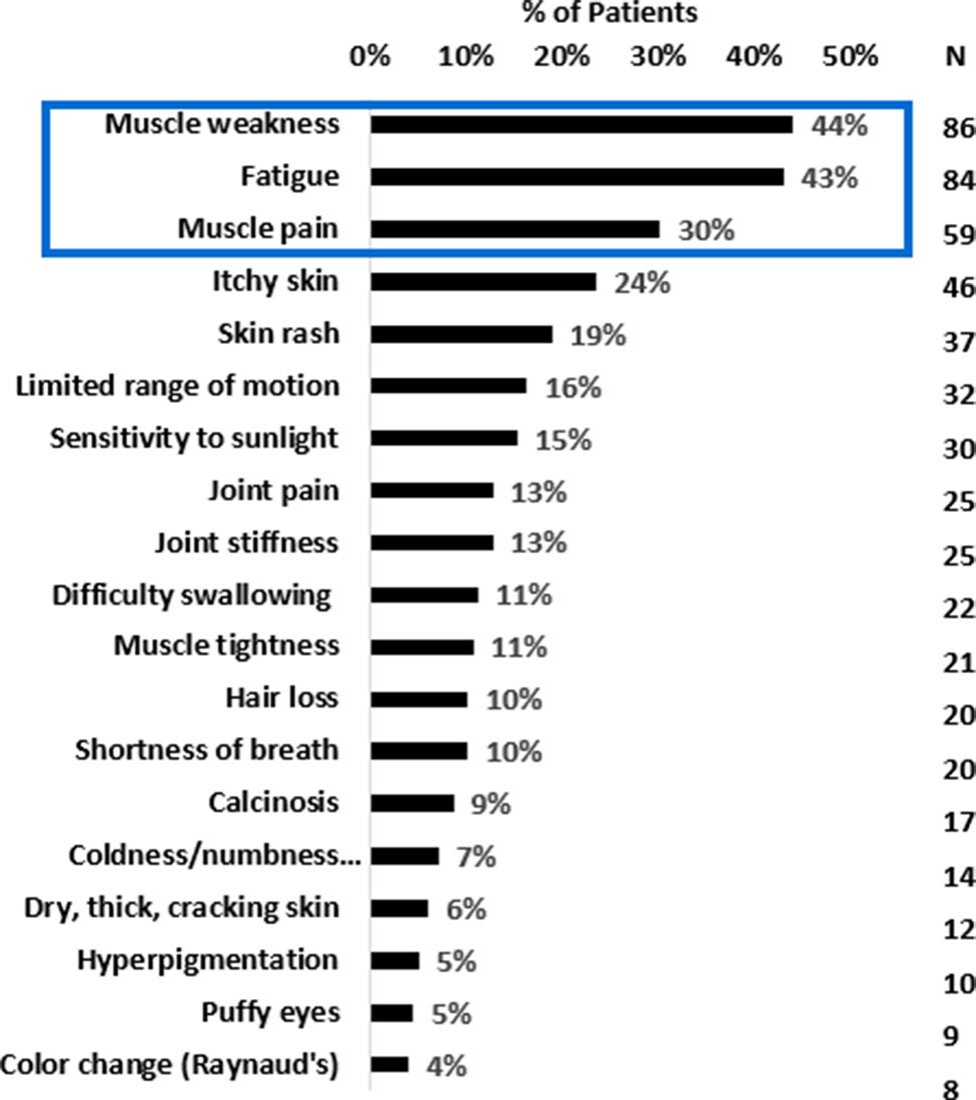
Muscle weakness and pain and fatigue are the three most bothersome symptoms reported by DM patients

At the time of completing the survey, 81% (158/195) of the respondents were experiencing muscle symptoms, 79% (154/195) were experiencing skin symptoms, and 32% (62/195) were experiencing other symptoms related to DM. Overall, the three most bothersome symptoms reported by respondents were muscle weakness (44%, 86/195), fatigue (43%, 84/195), and muscle pain (30%, 59/195) (Fig. 3). Interestingly, participants who rated the severity of their DM symptoms as moderate, severe, or very severe were significantly more likely to report muscle pain (*p* = 0.007) and limited range of motion (*p* = 0.0273) as one of the top three most bothersome symptoms while participants who rated the severity of their DM symptoms as mild were significantly more likely to report skin rash (*p* = 0.0059) and skin sensitivity to light (*p* = 0.0243) as one of the top three most bothersome symptoms. However, these values were not statistically significant after correction for multiple comparisons due to the number of most bothersome symptom options.

Respondents were also asked to rate the current severity of the remaining symptoms (i.e., not ones they selected as the most bothersome) using the following severity descriptors: not experiencing (symptom) today, mild, moderate, severe, very severe, and have never experienced this symptom. Across all 19 symptoms, fatigue was the top symptom respondents rated as severe (23%, 45/195) or very severe (9%, 18/195). Muscle weakness was the top symptom respondents rated as being of moderate severity (41%, 80/195) while skin rash (33%, 64/195) was the top symptom respondents rated as being of mild severity. The symptoms respondents most reported as “not experiencing today” were difficulty swallowing (45%, 88/195), puffy eyes (40%, 78/195), and color change in hands, fingers, toes, feet (40%, 78/195). The symptoms respondents most reported as “having never experienced” were calcinosis (39% 76/195) and color change in hands, fingers, feet, and toes (29%, 57/195). However, these infrequent symptoms were more likely to have been reported as severe.

#### Comorbidities

When asked about comorbidities, 30% (59/195) reported having none of the 11 listed comorbidities (cardiovascular disease, interstitial lung disease, rheumatoid arthritis, other pulmonary disease, Sjogren’s syndrome, cancer, diabetes, lupus, psoriasis, scleroderma, and psoriatic arthritis). The three most common comorbidities reported were cardiovascular disease (27%, 53/195), interstitial lung disease (22%, 43/195), and rheumatoid arthritis (17%, 33/195) (Additional File 1 Figure S1).

#### Impact on physical ability

A total of 10% (19/195) of respondents reported requiring assistance from another person and/or use of an assistive device to walk because of their DM. All 19 respondents used assistive devices at the time of the survey, including cane/crutches (74%, 14/19), walker (74%, 14/19), wheelchair (63%, 12/19), electric scooter (5%, 1/19), rollator (5%, 1/19), and service dog (5%, 1/19). Although most of the respondents were able to walk independently, more than half of all respondents reported that DM limited their ability to climb stairs (63%, 123/195) and to perform their usual daily activities (65%, 127/195).

#### Impact on social and mental health

**Fig. 4:**
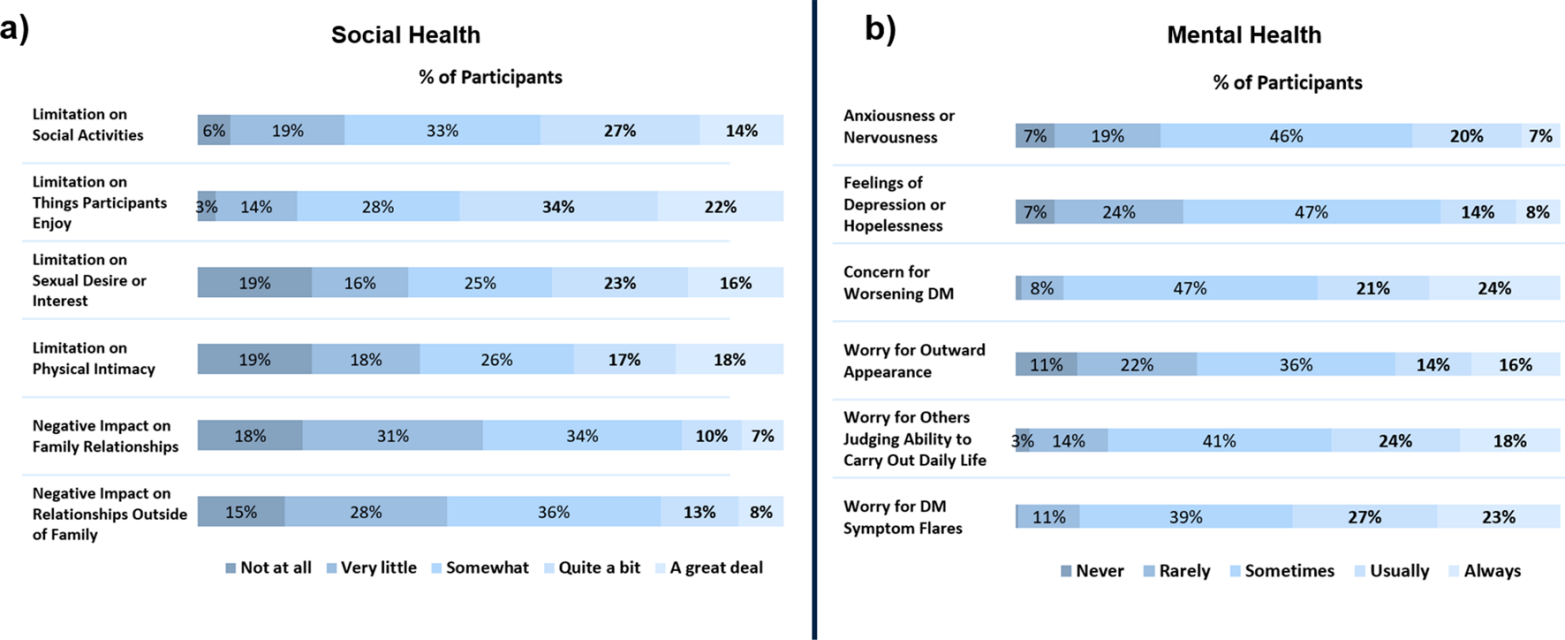
DM Impact on Social and Mental Health. (*a*) Impact on DM patients’ social health. For limitation on social activities, 11, 38, 65, 53 and 28 respondents selected “not at all”, “very little”, “somewhat”, “quite a bit” and “a great deal”, respectively. For limitation on things participants enjoy, 6, 27, 54, 66 and 42 respondents selected “not at all”, “very little”, “somewhat”, “quite a bit” and “a great deal”, respectively. For limitations on sexual desire, 38, 32, 48, 49 and 32 respondents selected “not at all”, “very little”, “somewhat”, “quite a bit” and “a great deal”, respectively. For limitations on physical intimacy, 38, 36, 51, 34 and 36 respondents selected “not at all”, “very little”, “somewhat”, “quite a bit” and “a great deal”, respectively. For negative impact on family relationships, 35, 60, 66, 20 and 14 respondents selected “not at all”, “very little”, “somewhat”, “quite a bit” and “a great deal”, respectively. For negative impacts on relationships outside the family, 29, 54, 71, 26 and 15 respondents selected “not at all”, “very little”, “somewhat”, “quite a bit” and “a great deal”, respectively. **(b)** Impact on DM patients’ mental health. For anxiousness, 14, 38, 90, 39 and 14 respondents selected respondents selected “never”, “rarely”, “sometimes”, “usually” or “always”, respectively. For depression, 14, 46, 42, 27 and 16 respondents selected “never”, “rarely”, “sometimes”, “usually” or “always”, respectively. For concern of worsening DM, 2, 15, 91, 40 and 47 respondents selected “never”, “rarely”, “sometimes”, “usually” or “always”, respectively. For worry for outward appearance, 22, 43, 71, 27 and 32 respondents selected “never”, “rarely”, “sometimes”, “usually” or “always”, respectively. For worry for others judging ability to carry out tasks, 5, 28, 80, 46 and 36 respondents selected “never”, “rarely”, “sometimes”, “usually” or “always”, respectively. For worry for DM flares, 1, 22, 76, 52 and 44 respondents selected “never”, “rarely”, “sometimes”, “usually” or “always”, respectively

Overall, DM had a negative impact on the respondents’ relationship with family and people outside the family (Fig. 4a). Approximately 51% (100/195) of respondents indicated that DM had a moderate (“somewhat”) to major (“a great deal”) negative impact on their relationships with family. Similarly, 57% (112/195) of respondents reported a moderate to major negative impact on relationships with people outside their family. DM also limited respondents’ sexual desire and ability to engage in physically intimate relationships with 64% (125/195) and 62% (121/195) reporting that they were “somewhat” to a “great deal” limited in their sexual desire and ability to engage in intimate relationships, respectively. Furthermore, when asked if DM limited the respondents’ ability to engage in social activities or “to do the things they enjoy,” almost all reported their social activities with family, friends, and neighbors to be limited “somewhat” to “a great deal” (75%, 146/195), while 83% (162/195) reported their ability to do the things they enjoyed were limited “somewhat” to a “great deal.”

Almost all respondents experienced some form of mental stress because of DM (Fig. 4b). Overall, 83% (162/195) reported having felt anxious or nervous or having felt down, depressed or hopeless either “sometimes,” “usually,” or “always” due to DM. Almost half of respondents (49%, 96/195) reported being “usually” or “always” worried about symptom flares and 45% (87/195) reported being “usually” or “always” worried about their DM getting worse. When respondents were asked if they were worried about DM limiting their ability to carry out daily activities, again a majority (83%, 162/195) responded “sometimes,” “usually,” or “always.”

### Impact on work

In this study population, 53% (103/195) were employed in some capacity, including 34% (67/195) working full time, 8% (16/195) working part-time, 4% (7/195) self-employed, and 7% (13/195) who were working but for no pay. There were 47 respondents (24%) on disability– 21% (41/195) on disability due to DM and 3% (6/195) due to another condition—and 5 respondents (3%) in school. When respondents were asked if they ever had to change their paid job status due to their DM (i.e., going from a full-time to a part-time position, going from working to disability, early retirement, etc.), a little less than half (49%, 95/195) responded “yes”.

**Fig. 5:**
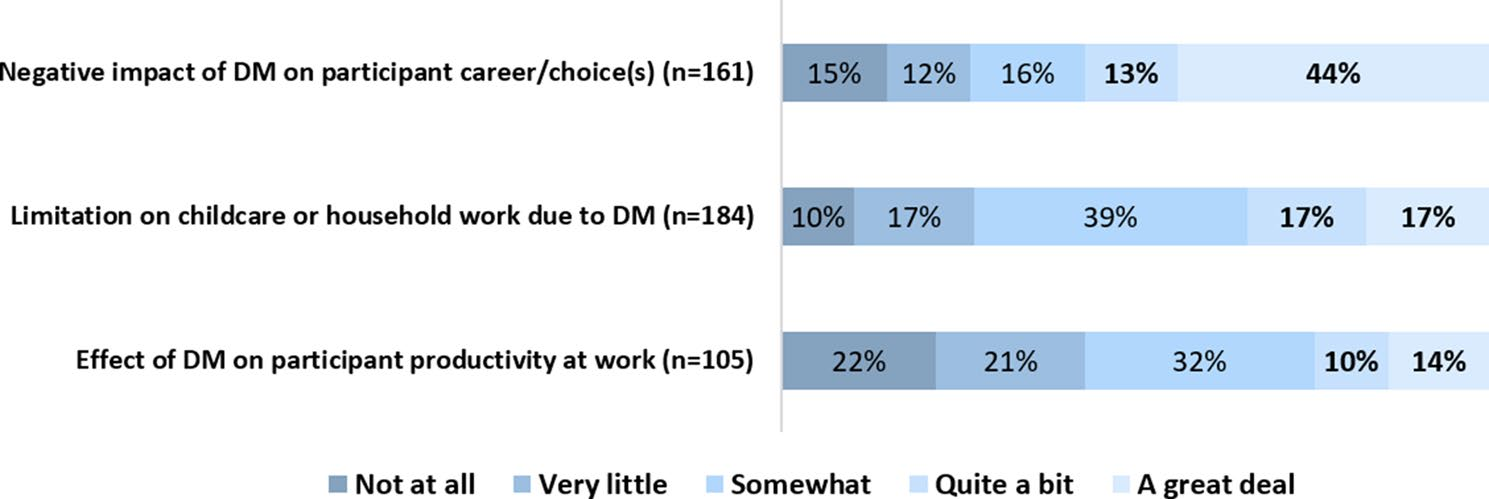
DM has a significant impact on participant’s productivity and ability to provide childcare. Of 161 respondents, 24, 19, 26, 21, 71 said DM had a negative impact on their career “not at all”, “very little”, “somewhat”, “quite a bit”, and “a great deal”, respectively. Of 184 respondents, 18, 31, 72, 31, and 31 said DM limited their childcare or household work “not at all”, “very little”, “somewhat”, “quite a bit”, and “a great deal”, respectively. Of 105 respondents, 23, 22, 34, 11, and 15 said DM affected their productivity at work DM “not at all”, “very little”, “somewhat”, “quite a bit”, and “a great deal”, respectively

In the 90 respondents who worked for pay, 29% (26/90) indicated that DM affected their productivity at work either “quite a bit” or “a great deal” in the past seven days with an average amount of three hours of missed work (Fig. 5). Interestingly, most respondents working for pay (50%, 45/90) felt that their employer accommodated their DM (i.e., flexible work schedules, easily reached work locations, work at home options, special workstation including chairs and ergonomic options). On the other hand, excluding the 34 people who checked “Not applicable”, 44% (71/161) of the remaining respondents reported that DM had a significant negative impact on their careers and/or career choices (Fig. 5).

With respect to work around the house and childcare, approximately a third (32%) answered “quite a bit” or “a great deal” when asked how much their DM limited their ability to care for their children or do household work in the past week (Fig. 5). For all 195 respondents, an average of four hours of childcare or housework around the home were missed in the past seven days.

### Satisfaction with care and support

The percentage of respondents currently receiving disability assistance for DM was 21% (41/195). More than half of the respondents have never received any of the other listed support services, ranging from 65% (127/195) having never received mental health support to 90% (176/195) having never received other types of financial aid. Except for disability assistance, less than 15% of the remaining respondents were receiving any of the other five health /social services (financial aid, paid homecare support, mental health support, home health support, healthcare management support).

When respondents were asked how satisfied they were with the level of community support services offered to persons living with DM, the majority (87%, 170/195) were either not at all, very little, or somewhat satisfied with the services offered indicating there is significant room for improvement in providing community services to the DM community. Additionally, when respondents were asked how often they engaged in discussions with other persons living with DM (i.e., online chat, social media, patient support groups), most (60%, 117/195) indicated that they never (“not at all”) or seldom (“very little”) engaged in discussions.

### Medication and management

**Fig. 6:**
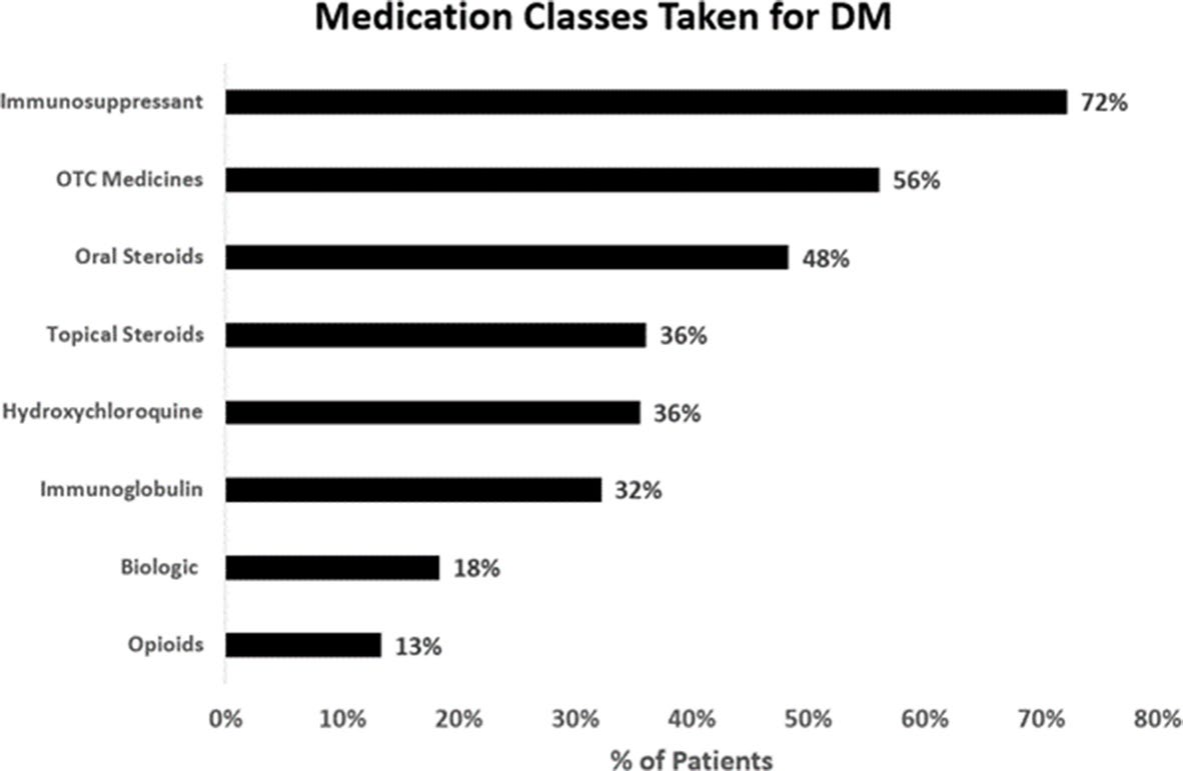
Medication Classes Taken by DM Patients. Among the 180 individuals that reported taking medication for DM, 72% (130/180) were taking immunosuppressants, 56% (101/180) were taking over the counter anti-inflammatory or pain medicines, 48% (86/180) were taking oral steroids, 36% (65/180) were taking topical steroids, 36% (65/180) were taking hydroxychloroquine, 32% (58/180) were taking immunoglobulin, 18% (32/180) were taking biologic, and 13% (23/180) were taking opioids. 23% (41/180) were also taking medications/supplements that were not listed (data not shown on graph)

Most respondents (92%, 180/195) were taking medication for DM (Fig. 6). Of those 180 patients taking medications, 92% (166/180) were on 2 + medications. Among the patients currently taking medications, 52% (94/180) reported that the medication caused “some” to a “great deal of stress” for them and their family. Of note, 62% (76/122) of patients with self-reported moderate to very severe DM were less than satisfied with their medications and were significantly more likely to be somewhat dissatisfied to very dissatisfied with their medication than, patients with self-reported mild DM, who were significantly more likely to be somewhat satisfied to very satisfied with their medications (*p* = 0.0039). Therefore, the unmet need is significantly higher in patients with greater disease severity.

A little less than half of the respondents (45%, 87/195) were on oral corticosteroids (OCS) at the time of filling out the survey. Considering that corticosteroids are associated with muscle weakness [19], an analysis was conducted to assess whether muscle weakness, one of the three most selected bothersome symptoms, was correlated with OCS use. There was no statistically significant difference in the likelihood of experiencing muscle weakness or the other two most selected bothersome symptoms, muscle pain and fatigue, based on OCS use.

Among the 15 respondents who were not taking medications, the reasons for stopping their last medication include side effects (40%, 6/15), efficacy (20%, 3/15), loss of efficacy (13%, 2/15), had to take the medication too often (13%, 2/15), burdensome cost (13%, 2/15), disease went dormant/medication worked (7%, 1/15), insurance issues (7%, 1/15), and symptom resolution (7%, 1/15).

## Discussion

The purpose of this survey was to capture the heterogenous patient experience of individuals living with DM with consideration for their mental, physical, social, and medication burdens. Indeed, there was a diverse range of patients most bothersome symptoms, severity of disease, physical and mental burden, and satisfaction with care and treatment. However, one prevailing observation was that those with the longest disease duration reported the most severe symptoms. The patients with greater disease severity were also the ones with increased social and physical burden, increased use of medications and, importantly, increased dissatisfaction with medication options. Of note, a greater proportion of DM patients with severe disease were taking opioids for symptom management than might have previously been appreciated. Additionally, patients who were not taking any medication for symptom management at the time of the survey typically discontinued due to side effects or lack of efficacy. Notably, only 14% of the 15 patients who discontinued medication stopped due to symptoms resolving, indicating a substantial failure of current medications to adequately manage DM patient symptoms. Unsurprisingly, there were also high rates of self-reported comorbidities, which align with previous findings of increased rates of cardiovascular risk [20], interstitial lung disease [21], and rheumatoid arthritis [22] in DM patients compared to matched controls. All this data provides explanation to why the greatest percentage of participants rated their overall health as only “fair”. In fact, only two respondents reported having overall excellent health. Upon further examination, these data could be an outlier or a data entry error as these two respondents also reported experiencing severe dermatomyositis symptoms.

It would be expected that the most bothersome symptoms would also be rated as the most severe. However, when looking at the top three symptoms (muscle weakness, fatigue, muscle pain), more than half of the respondents who selected muscle weakness went on to rate the severity of their muscle weakness as mild or moderate (20% − 17/86 and 59% − 51/86, respectively) with only 21% (18/86) rating their muscle weakness as severe or very severe. Similarly, respondents who selected muscle pain rated the severity of their muscle pain as mild or moderate (15% − 9/59 and 56% − 33/59, respectively) with only 28% (17/59) rating the severity of their muscle pain as severe or very severe. Therefore, bothersome symptoms do not necessarily have to be severe, as even mild or moderate symptoms can significantly impact quality of life. Overall, these results support the progressive, debilitating nature of the disease and the continued unmet need in the DM patient population.

Although there was high variation in age and disease severity of survey respondents, the survey was limited to U.S. respondents, which limits the true diversity of the patient experience. Additionally, only 9% (18/195) of the survey respondents self-identified as Black or African American while 82% (160/195) self-identified as White, which also limited out ability to understand the effect of race on DM patient experience. TMA does not ask about members race so whether this disproportionality is due to the make-up of TMA e-mail list from which DM patients were invited to participate, the effect of the survey’s inclusion criteria, one race’s propensity towards participating in research over others, or another reason is unknown. Most participants were also highly educated and insured. It is possible that participants with a higher degree of education are more likely to be financially stable and more likely to be involved in support organizations such as TMA. It is also possible that less affluent individuals may not have had access to an online survey due to technological barriers. Since DM causes significant financial burden that is likely to increase with disease severity due to increased medication burden and impact on work, it is possible that patients with more severe disease may be less able to participate in this online survey. Therefore, efforts to increase diversity in respondents, economically, racially, and geographically should be the goal of any future survey because DM has been estimated to have a 3-fold greater incidence in Black individuals than in White individuals [23].

Some steps that may be taken to increase representation of different races, education levels, and income levels in future surveys include sending the surveys to more support organizations, advertising on social media, or partnering with DM physicians directly to provide surveys to patients. The latter option would allow for DM patients to participate in more rural or underrepresented areas as well as provide the opportunity for paper versions of the survey to be provided during clinic visits, eliminating any technology barriers. Partnering directly with physicians to recruit DM patients also addresses another limitation of this study regarding the lack of diagnosis verification, though this option would also significantly increase the time needed for enough patients to complete the survey. Finally, conducting the survey in a larger sample size would allow for more robust correlation analyses between various aspects of patients’ DM experience.

Nevertheless, this survey provided a more in-depth and comprehensive look into the diverse DM patient perspective than has been previously reported in the literature. Despite a wide range of symptom management options, including corticosteroids and the recently approved intravenous immunoglobin [24], patients are still reporting debilitating physical and mental symptoms and are dissatisfied with their management options. Notably, none of the currently approved medications for DM have been proven to target the pathogenesis of the disease. Therefore, continued development of targeted therapies is essential to address the unmet need of DM patients.

## Conclusion

Results from this survey have demonstrated that DM significantly impacts patients’ QoL with widespread effects on physical function, mental and social health, and financial stability. Demonstrated dissatisfaction with available therapies elucidate the unmet need for novel, efficacious, targeted medications to further improve DM patients’ QoL. In addition to clinical research and medication options, this survey has demonstrated that patients are dissatisfied with community and financial support. By highlighting these deficiencies, advocacy groups like TMA can develop improved methods of communication with DM patients of different ages, aid patients in receiving mental health counseling, and advocate for policy changes that increase and/or improve the level of financial support that DM patients receive. Although DM is a rare disease, it is important to consider the varied experiences of the population and provide resources and support that can be tailored to a patient’s individual burden, and this survey was the next, important step in increasing understanding of what the DM community needs: increased financial support, mental health awareness, and treatment options that go beyond temporary symptom management.

## Electronic supplementary material

Below is the link to the electronic supplementary material.

Supplementary Material 1

## Data Availability

Data are available upon reasonable request to the corresponding author.

## Abbreviations

DM: Dermatomyositis
IRB: Institutional Review Board
QoL: Quality of Life
OCS: oral corticosteroid
PROMIS: Patient–Reported Outcomes Measurement Information System
TMA: The Myositis Association
