# Supplementary material for: Patient reported physical function, mental health, and treatment patterns in dermatomyositis: survey results from a cross-sectional study of adult dermatomyositis patients
Source: BMC Rheumatol. 2025 Feb 24;9:23. doi: 10.1186/s41927-025-00458-2 (PMC11849329; doi:10.1186/s41927-025-00458-2)
Supplement: Supplementary file 1 — Supplementary Material 1 [file 41927_2025_458_MOESM1_ESM.docx]

**Supplement for:**

**Patient Reported Physical Function, Mental Health, and Treatment Patterns in Dermatomyositis: Survey Results from a Cross-Sectional Study of Adult Dermatomyositis Patients**

***Lisa Christopher-Stine^1^, Julie J. Paik^1^, Alexandra S. Goriounova^2^, and Paul N. Mudd Jr.^2^**

**Affiliations:** ^1^Division of Rheumatology, Department of Medicine, Johns Hopkins University School of Medicine, Baltimore, MD, USA, and ^2^Priovant Therapeutics Inc., New York, NY, USA


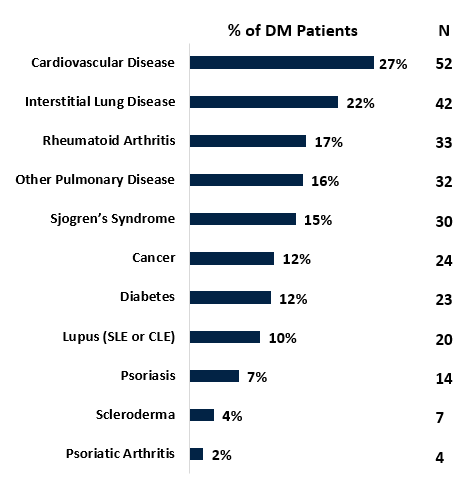


**Supplementary Figure S1: Self-reported comorbidities of respondents.** 59/195 (30%) of patients reported having none of the listed comorbidities (data not shown). SLE = systemic lupus erythematous; CLE = cutaneous lupus erythematous

**Supplementary Table S1: Assessment of Survey Consistency and Reliability Using Cronbach’s Alpha.** Questions that related to how a participant feels and functions were selected for reliability analysis. Demographic, medication, symptom selection, mobility aid, support services, and yes/no questions were excluded.

| **Category** | **Cronbach’s Alpha (95% CI)** |
| --- | --- |
| Overall Health and DM Symptom Severity: Q11 Q12 | 0.61 (0.46, 0.72) |
| Social Domain: Q21 Q22 Q23 Q24 Q25 Q26 | 0.89 (0.86, 0.91) |
| Emotional Domain: Q27 Q28 Q29 Q30 Q31 Q32 | 0.87 (0.83, 0.90) |
| Functional Domain: Q39 Q40 Q42 | 0.86 (0.80, 0.91) |
| Overall Health and Social Domain: Q11 Q21 Q22 Q23 Q24 Q25 Q26 | 0.89 (0.86, 0.91) |
| Overall Health and Emotional Domain: Q11 Q27 Q28 Q29 Q30 Q31 Q32 | 0.86 (0.83, 0.89) |
| Overall Health and Functional Domain: Q11 Q39 Q40 Q42 | 0.85 (0.78, 0.89) |
| DM Symptom Severity and Social Domain: Q12 Q21 Q22 Q23 Q24 Q25 Q26 | 0.88 (0.85, 0.91) |
| DM Symptom Severity and Emotional Domain: Q12 Q27 Q28 Q29 Q30 Q31 Q32 | 0.85 (0.81, 0.88) |
| DM Symptom Severity and Functional Domain: Q12 Q39 Q40 Q42 | 0.86 (0.81, 0.90) |
| All | 0.94 (0.92, 0.96) |

**Supplementary Data S1: Full Questionnaire**

|  | | | | Response | |
| --- | --- | --- | --- | --- | --- |
| 1. Please state your age (in years). | | | |  | |
| 1. In what State do you currently live? | | | | | |
|  | I do not live in the US. | | | | |
|  | Alabama (AL) |  | Alaska (AK) |  | Arizona (AZ) |
|  | Arkansas (AR) |  | California (CA) |  | Colorado (CO) |
|  | Connecticut (CT) |  | Delaware (DE) |  | Florida (FL) |
|  | Georgia (GA) |  | Hawaii (HI) |  | Idaho (ID) |
|  | Illinois (IL) |  | Indiana (IN) |  | Iowa (IA) |
|  | Kansas (KS) |  | Kentucky (KY) |  | Louisiana (LA) |
|  | Maine (ME) |  | Maryland (MD) |  | Massachusetts (MA) |
|  | Michigan (MI) |  | Minnesota (MN) |  | Mississippi (MS) |
|  | Missouri (MO) |  | Montana (MT) |  | Nebraska (NE) |
|  | Nevada (NV) |  | New Hampshire (NH) |  | New Jersey (NJ) |
|  | New Mexico (NM) |  | New York (NY) |  | North Carolina (NC) |
|  | North Dakota (ND) |  | Ohio (OH) |  | Oklahoma (OK) |
|  | Oregon (OR) |  | Pennsylvania (PA) |  | Rhode Island (RI) |
|  | South Carolina (SC) |  | South Dakota (SD) |  | Tennessee (TN) |
|  | Texas (TX) |  | Utah (UT) |  | Vermont (VT) |
|  | Virginia (VA) |  | Washington (WA) |  | West Virginia (WV) |
|  | Wisconsin (WI) |  | Wyoming (WY) |  | Washington DC |

| 1. Sex assigned at birth | |
| --- | --- |
|  | Male |
|  | Female |

| 1. Ethnicity/Race (Please select only one option with which you most closely identify) | |
| --- | --- |
|  | American Indian or Alaska native |
|  | Asian |
|  | Black or African American |
|  | Hispanic, Latino, or Spanish Origin |
|  | Middle Eastern or North African |
|  | Native Hawaiian or Other Pacific Islander |
|  | White |
|  | Two or more ethnicities / races |
|  | Other Race or Ethnicity |

| 1. The highest level of education that you have completed | |
| --- | --- |
|  | Less than high school |
|  | High school graduate |
|  | Some college, no degree |
|  | Associates degree |
|  | Bachelor’s degree |
|  | Postgraduate degree |

| 1. Current health insurance coverage status. *Please check all that apply.* | |
| --- | --- |
|  | Private/Commercial health insurance |
|  | Public/Government health insurance (e.g., Medicare, Medicaid) |
|  | Uninsured |

| 1. Has a health care provider (e.g., doctor, nurse practitioner, physician assistant) ever told you that you had juvenile dermatomyositis? | |
| --- | --- |
|  | Yes |
|  | No |

| 1. How long in years have you been experiencing dermatomyositis symptoms? | |
| --- | --- |
|  | Less than 3 years |
|  | 3 to 5 years |
|  | >5 years to 10 years |
|  | More than 10 years |

|  | Response |
| --- | --- |
| 1. In what year were you first told by a health care provider (e.g., doctor, nurse practitioner, physician assistant) that you had dermatomyositis? *Please estimate as best as you can.* | A dropdown menu displaying all years from 1946 – 2022 |
| 1. In what month of ___ (the year selected in Q15) were you first told by a health care provider (e.g., doctor, nurse practitioner, physician assistant) that you had dermatomyositis? *If you don’t recall the month, it is okay to skip this question.* | A dropdown menu displaying all months from January – December |

|  | Mild | Moderate | Severe | Very severe |
| --- | --- | --- | --- | --- |
| 1. How would you describe the severity of your overall dermatomyositis symptoms today? |  |  |  |  |

|  | Excellent | Very good | Good | Fair | Poor |
| --- | --- | --- | --- | --- | --- |
| 1. How would you describe your overall health today? |  |  |  |  |  |

| 1. Please select up to three (3) dermatomyositis symptoms that bother you the most today. | | | | | |
| --- | --- | --- | --- | --- | --- |
|  | Difficulty swallowing |  | Muscle weakness |  | Muscle pain |
|  | Muscle tightness |  | Joint pain |  | Joint stiffness |
|  | Skin rash |  | Hyperpigmentation (dark spots / patches on the skin) |  | Itchy skin |
|  | Hair loss |  | Dry, thick, cracking skin (e.g., mechanic's hand) |  | Skin sensitivity to sunlight |
|  | Shortness of breath |  | Fatigue |  | Puffy eyes |
|  | Discomfort from hard calcium deposits under skin (calcinosis) |  | Limited range of motion, including difficulty lifting your arms |  | Coldness/numbness/tingling of hands, fingers, feet, or toes (Raynaud's phenomenon) |
|  | Color change in hands, fingers, feet, or toes (blue/red/white) (Raynaud's phenomenon) |  |  |  |  |

| 1. (For the symptoms selected in Q13) Please select the response that best describes how severe these dermatomyositis symptoms are for you today. | | | | |
| --- | --- | --- | --- | --- |
| Symptom | Mild | Moderate | Severe | Very severe |
| Symptom 1 selected in Q13 |  |  |  |  |
| Symptom 2 selected in Q13 |  |  |  |  |
| Symptom 3 selected in Q13 |  |  |  |  |

| 1. (For the symptoms not selected in Q13) Please select the response that best describes how severe the following other dermatomyositis symptoms are for you today.  - If you have never experienced a listed symptom, please select "Haver never experienced this symptom". - If you have experienced a symptom, but it is not affecting you today, please select “Not experiencing today” for that symptom. | | | | | | |
| --- | --- | --- | --- | --- | --- | --- |
| Symptom | Not at all (today) | Mild | Moderate | Severe | Very severe | Have never experienced this symptom |
| Symptoms not selected in Q13 |  |  |  |  |  |  |

|  | | Yes | No |
| --- | --- | --- | --- |
| 1. Are you currently able to walk without the help of another person and without use of an assistive device? | |  |  |
| 1. (If answer ‘no’ to Q16) Are you currently using assistive devices (e.g., cane, walker, crutches, leg braces, wheelchair, etc.)? | |  |  |
| 1. (If answer ‘yes’ to Q17) Please specify all assistive devices you use to help you walk: | | | |
|  | Wheelchair | | |
|  | Cane/crutches | | |
|  | Leg braces | | |
|  | Walker | | |
|  | Other (please specify): | | |

|  | Yes | No |
| --- | --- | --- |
| 1. Does dermatomyositis limit your ability to climb stairs? |  |  |
| 1. Does dermatomyositis limit your ability to perform your usual daily activities? |  |  |

Please select the response that best describes the impact of dermatomyositis on your life.

|  | Not at all | Very little | Somewhat | Quite a bit | A great deal |
| --- | --- | --- | --- | --- | --- |
| 1. Does dermatomyositis limit your normal social activities with family, friends, or neighbors? |  |  |  |  |  |
| 1. Does dermatomyositis limit your ability to do the things that you enjoy? |  |  |  |  |  |
| 1. Does dermatomyositis limit your sexual desire or interest? |  |  |  |  |  |
| 1. Does dermatomyositis limit your ability to have physically intimate relationships? |  |  |  |  |  |

|  | Not at all | Very little | Somewhat | Quite a bit | A great deal |
| --- | --- | --- | --- | --- | --- |
| 1. How much does dermatomyositis negatively impact your relationships with family? |  |  |  |  |  |
| 1. How much does dermatomyositis negatively impact your relationships with people outside your family? |  |  |  |  |  |

| How often do you: | Never | Rarely | Sometimes | Usually | Always |
| --- | --- | --- | --- | --- | --- |
| 1. Feel anxious or nervous due to dermatomyositis? |  |  |  |  |  |
| 1. Feel down, depressed, or hopeless due to dermatomyositis? |  |  |  |  |  |
| 1. Worry about your dermatomyositis getting worse? |  |  |  |  |  |
| 1. Worry about what others think of your outward appearance due to dermatomyositis? |  |  |  |  |  |
| 1. Worry about what others think of your limited ability to carry out your daily activities due to dermatomyositis? |  |  |  |  |  |
| 1. Worry about dermatomyositis symptom flares? |  |  |  |  |  |

| 1. Please state your current working status. *Please check all that apply.* | |
| --- | --- |
|  | Working (Full time) for pay |
|  | Working (Part time) for pay |
|  | Working (Self-employed) for pay |
|  | Not working for pay |
|  | Retired |
|  | On disability due to dermatomyositis |
|  | On disability due to other condition |
|  | Student in school |

|  | Yes | No |
| --- | --- | --- |
| 1. Have you ever had to change your paid job status due to dermatomyositis (e.g., from full-time to part-time, from part-time to disability, early retirement, etc.)? |  |  |

|  | Hours |
| --- | --- |
| 1. How many hours do you currently do paid work in a typical week? *If you currently do not do paid work, please skip this question.* |  |
| 1. In the past 7 days, how many hours of the (the number of hours entered in Q35)-hour paid work did you miss due to your dermatomyositis (including hours missed on sick days, times you started late or stopped early due to dermatomyositis)? *If you currently do not do paid work, please skip this question.* |  |
| 1. How many hours do you spend on childcare or household work around the home in a typical week? |  |
| 1. In the past 7 days, how many hours of the (the number of hours entered in Q37)-hour childcare or household work around the home were you unable to do due to your dermatomyositis? |  |

Please select the response that best describes the impact of dermatomyositis on your work.

|  | Not at all | Very little | Somewhat | Quite a bit | A great deal | N/A |
| --- | --- | --- | --- | --- | --- | --- |
| 1. In the past 7 days, how much did your dermatomyositis affect your productivity at paid work? |  |  |  |  |  |  |
| 1. In the past 7 days, how much did dermatomyositis limit your ability to do childcare or household work around the home? |  |  |  |  |  |  |
| 1. How well do you feel that your employer accommodates people living with dermatomyositis (e.g., flexible work schedules, easily reached work locations, work at home options, special workstation including chairs and ergonomic options)? |  |  |  |  |  |  |
| 1. Do you feel that dermatomyositis has negatively impacted your career and/or career choices? |  |  |  |  |  |  |

| Have you ever received: | Never received | Received in the past | Currently receiving |
| --- | --- | --- | --- |
| 1. Disability assistance for dermatomyositis |  |  |  |
| 1. Other types of financial aid (e.g., charity, support groups) for dermatomyositis |  |  |  |
| 1. Healthcare management support (e.g., case manager, transportation, appointment management) due to your dermatomyositis. |  |  |  |
| 1. Home health support (e.g., part-time/intermittent skilled nursing services, home healthcare worker, and rehabilitation therapy at home) due to your dermatomyositis. |  |  |  |
| 1. Other forms of paid home care support (e.g., for household tasks, childcare) due to your dermatomyositis. |  |  |  |
| 1. Mental health support (e.g., psychiatrist, psychologist, counseling, helpline) due to your dermatomyositis. |  |  |  |

Please select the response that best describes services and engagement for persons living with dermatomyositis.

|  | Not at all | Very little | Somewhat | Quite a bit | A great deal |
| --- | --- | --- | --- | --- | --- |
| 1. Overall, how satisfied are you with the level of support services that are offered to persons living with dermatomyositis? |  |  |  |  |  |
| 1. How often do you engage in discussions with other persons living with dermatomyositis (e.g., online chat, social media, patient support groups)? |  |  |  |  |  |

| 1. Are you currently being treated/currently taking medication for dermatomyositis? | |
| --- | --- |
|  | Yes |
|  | No |
|  | Never treated/never taken medication for dermatomyositis |

| 1. (If answer ‘yes’ to Q51) Please select all of the classes of medications you are currently taking to treat your dermatomyositis. *Please check all that apply.* | |
| --- | --- |
|  | Oral Steroids (e.g., prednisone, dexamethasone) |
|  | Topical Steroids (e.g., triamcinolone acetonide, clobetasol propionate) |
|  | Hydroxychloroquine (e.g., Plaquenil®) |
|  | Immunosuppressant (e.g., methotrexate, Rasuvo®, mycophenolate mofetil, azathioprine) |
|  | Biologic (e.g., Rituxan®, Avastin®, Humira®) |
|  | Immunoglobulin (IVIG, e.g., Gammagard Liquid®, Octagam 10%®) |
|  | Over the counter anti-inflammatory or pain medicines (e.g., Advil®, Aleve®, Tylenol® etc.) |
|  | Opioids (e.g., OxyContin®, Vicodin®) |
|  | Other (please specify): __________________________ |

| 1. (If answer ‘no’ to Q51) What was the class of medication that you were last taking to treat dermatomyositis? *Please check one.* | |
| --- | --- |
|  | Oral Steroids (e.g., prednisone, dexamethasone) |
|  | Topical Steroids (e.g., triamcinolone acetonide, clobetasol propionate) |
|  | Hydroxychloroquine (e.g., Plaquenil®) |
|  | Immunosuppressant (e.g., methotrexate, Rasuvo®, mycophenolate mofetil, azathioprine) |
|  | Biologic (e.g., Rituxan®, Avastin®, Humira®) |
|  | Immunoglobulin (IVIG, e.g., Gammagard Liquid®, Octagam 10%®) |
|  | Over the counter anti-inflammatory or pain medicines (e.g., Advil®, Aleve®, Tylenol® etc.) |
|  | Opioids (e.g., OxyContin®, Vicodin®) |
|  | Other (please specify): __________________________ |

| 1. (If answer ‘no’ to Q51) For the class of medication that you were last taking, what was the type of treatment? *Please check one.* | | | | | |
| --- | --- | --- | --- | --- | --- |
|  | Solid oral medication (e.g., tablets or capsules) |  | Liquid oral medication |  | Topical medication |
|  | Inhaled medication |  | Subcutaneous injection (injected through the skin) |  | Intravenous injection by a healthcare professional |
|  | Other (please specify): ________________ |  |  | | |

| 1. (If answer ‘no’ to Q51) Why did you stop this treatment? *Please select all options that apply.* | | | | | |
| --- | --- | --- | --- | --- | --- |
|  | Treatment did not work |  | Treatment took a long time to work |  | Treatment worked less after some time |
|  | Side effects |  | Had to take the treatment too often |  | How I had to take the medication (e.g., solid oral medication, liquid oral medication, subcutaneous injection, intravenous injection) |
|  | Location of treatment administration (e.g., at home, at the hospital) was inconvenient |  | Cost of treatment was too high |  | Other reasons (please specify): _______________ |

|  | Not at all | Very little | Some | Quite a bit | A great deal |
| --- | --- | --- | --- | --- | --- |
| 1. (If answer ‘yes’ to Q51) Does the cost of your current dermatomyositis treatment cause stress for you and/or your family? |  |  |  |  |  |

| 1. (If answer ‘yes’ to Q51) How would you rate the value of your current dermatomyositis treatment, given how much it costs? | |
| --- | --- |
|  | Extremely poor value |
|  | Poor value |
|  | Ok value |
|  | Good value |
|  | Extremely good value |

|  | Very Dissatisfied | Dissatisfied | Somewhat dissatisfied | Somewhat Satisfied | Satisfied | Very Satisfied |
| --- | --- | --- | --- | --- | --- | --- |
| 1. (If answer ‘yes’ to Q51) Taking all things into account, how satisfied or dissatisfied are you with your current dermatomyositis treatment? |  |  |  |  |  |  |

| 1. (If answer ‘yes’ to Q51) If you are currently taking solid oral medications, how do you take your medications? *Please select all options that apply.* | |
| --- | --- |
|  | I swallow the tablet/capsule whole |
|  | I crush the tablet/I open the capsule |
|  | I crush/mix the tablet/capsule with other food/drinks |
|  | I chew the tablet/capsule |
|  | I split the tablet/capsule into smaller sizes |
|  | Other |
|  | N/A (Not currently taking any solid oral medication) |

| 1. Has a health care provider (e.g., doctor, nurse practitioner, physician assistant) told you that you had any of the following conditions? *Please select all that apply.* | |
| --- | --- |
|  | Cancer |
|  | Cardiovascular disease (e.g., heart failure, hypertension) |
|  | Diabetes |
|  | Psoriasis |
|  | Scleroderma |
|  | Psoriatic arthritis |
|  | Rheumatoid arthritis |
|  | Sjogren’s syndrome |
|  | Systemic or cutaneous lupus erythematosus (SLE or CLE) |
|  | Interstitial lung disease |
|  | Other pulmonary disease (e.g., chronic obstructive pulmonary disease (COPD), asthma) |
|  | None of the above |
